# Exploring Reverse Sural Flap Necrosis in Lupus-like Syndrome: Challenges and Strategies in Lower Limb Reconstruction—A Case Presentation

**DOI:** 10.3390/medicina60122053

**Published:** 2024-12-13

**Authors:** Alessandra Ceccaroni, Roberto Cuomo, Paola Pentangelo, Antonioenrico Gentile, Caterina Marra, Warren Matthew Rozen, Ishith Seth, Bryan Lim, Carmine Alfano

**Affiliations:** 1 Plastic Surgery Unit, Department of Medicine, Surgery and Dentistry, University of Salerno, Baronissi, 84081 Salerno, Italy; ppentangelo@unisa.it (P.P.); ag.gentile4@gmail.com (A.G.); camarra@unisa.it (C.M.); calfano@unisa.it (C.A.); 2 Plastic and Reconstructive Surgery Division, Department of Medicine, Surgery and Neuroscience, Santa Maria alle Scotte Hospital, University of Siena, 53100 Siena, Italy; robertocuomo@outlook.com; 3 Department of Plastic Surgery, Peninsula Health, Melbourne, VIC 3199, Australia; warrenrozen@hotmail.com (W.M.R.); ishithseth1@gmail.com (I.S.); lim.bryan58@gmail.com (B.L.)

**Keywords:** reverse sural flap, lower limb reconstruction, soft tissue defect, lupus-like syndrome, reverse sural flap necrosis.

## Abstract

Soft tissue reconstruction in the lower limbs presents a significant challenge, particularly when addressing defects in the distal third of the leg, ankle, and foot. The reverse sural flap reliant on the perforating branches of the peroneal artery has emerged as a versatile option, offering a solution for patients for whom microsurgical techniques are not feasible. Despite its advantages, the procedure carries inherent risks, especially in populations with underlying conditions, such as venous insufficiency, cardiovascular disease, and diabetes, as well as in elderly patients, where the likelihood of flap necrosis is elevated. This report details a case of reverse sural flap necrosis in a patient with lupus-like syndrome, a complex scenario that underscores the need for meticulous preoperative assessment and planning. The case illustrates not only the technical considerations and challenges associated with the reverse sural flap but also the broader implications of systemic autoimmune disorders on postoperative outcomes. Through a comprehensive review of the literature, we explore the relationship between vascularization, autoimmune profiles, and the success of reverse sural flap procedures. We highlight the critical need for surgeons to adopt a holistic approach to patient evaluation, considering both local and systemic factors that may influence the viability of the flap and the overall reconstructive success.

## 1. Introduction

The reconstruction of soft tissue defects particularly in the distal leg, ankle, and foot poses a significant challenge in plastic and reconstructive surgery [1]. Among the various techniques available, the reverse sural artery flap (RSF) has emerged as a valuable option due to its reliability, simplicity, and the conservation of major arteries [1,2]. The reverse sural flap has been a subject of considerable interest in reconstructive surgery, particularly for soft tissue defects in the lower leg, ankle, and foot [3]. The reverse sural flap leverages the sural artery and venae comitans for vascular supply, making it a versatile choice for covering defects resulting from trauma, infections, and other aetiologies [4]. Donski et al. first introduced the concept of the distally based sural fasciocutaneous flap in 1983, specifically for coverage of the Achilles tendon. Subsequently, Masquelet et al. provided a comprehensive description of the anatomy of this flap in 1992 [2,3,4,5]. To date, there are doubts about performing the reverse sural flap in patients with venous insufficiency, cardiovascular diseases, and diabetes, as well as in elderly patients due to a high risk of flap necrosis [6]. However, complications, such as flap necrosis, can significantly impact the outcomes of this reconstructive approach [7]. Factors contributing to flap necrosis include venous congestion, inadequate perfusion, and patient-specific factors, such as underlying systemic diseases. Lupus-like syndrome, an autoimmune condition characterized by symptoms similar to systemic lupus erythematosus (SLE), presents an added complexity in flap surgery due to potential vasculopathy and impaired wound healing [8,9,10]. This case report and literature review focus on a unique case of reverse sural flap necrosis in a patient with lupus-like syndrome, underscoring the challenges and management strategies involved in such complex scenarios. The aim is to provide insights into the intricacies of performing RSF in patients with autoimmune conditions and analyze potential risk factors for flap failure.

## 2. Case Presentation

**Figure 1:**
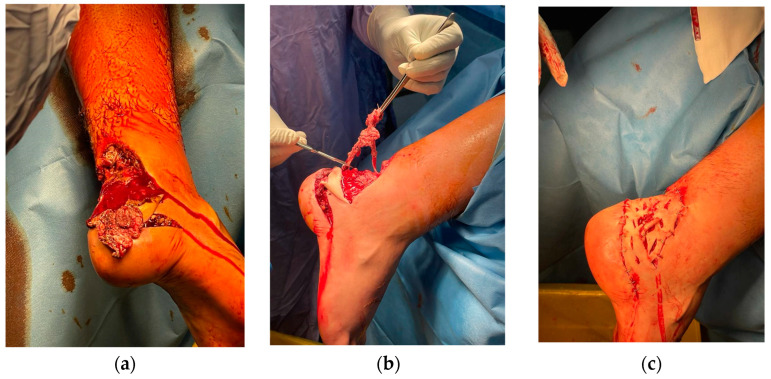
Preoperative view showing the extent of soft tissue loss (**a**); intraoperative scene of debridement process (**b**); application of skin graft derived from lesion area to cover tissue defect (**c**).

A 20-year-old healthy male was admitted to our tertiary trauma centre after sustaining a 7 × 7 cm² soft tissue defect on the right lower limb, specifically at the calcaneus and external malleolus, along with an Achilles tendon rupture from a motor vehicle accident. He was otherwise healthy, not smoking and not taking any medications. Initial management included immediate debridement, coverage of the soft-tissue defect with a skin graft sourced from the lesion area, and application of negative pressure wound therapy (NPWT) (Figure 1a–c).

**Figure 2:**
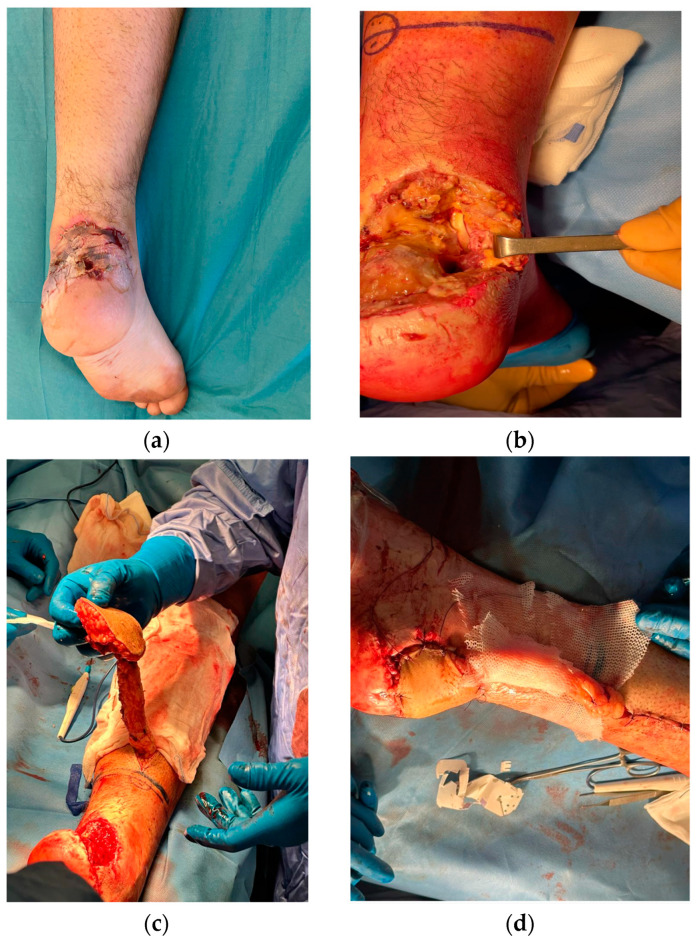
Necrosis manifestation on skin graft, observed 10 days post-operation (**a**); Achilles tendon reconstructed using flexor hallucis longus tendon (**b**); process of elevating the reverse sural flap for reconstruction (**c**); coverage of the soft tissue defect with the reverse sural flap (**d**).

However, 10 days post-operation, necrosis and infection prompted a secondary surgical intervention for Achilles tendon reconstruction using the flexor hallucis longus tendon and a reverse sural flap to cover the soft-tissue defects (Figure 2a–d).

**Figure 3:**
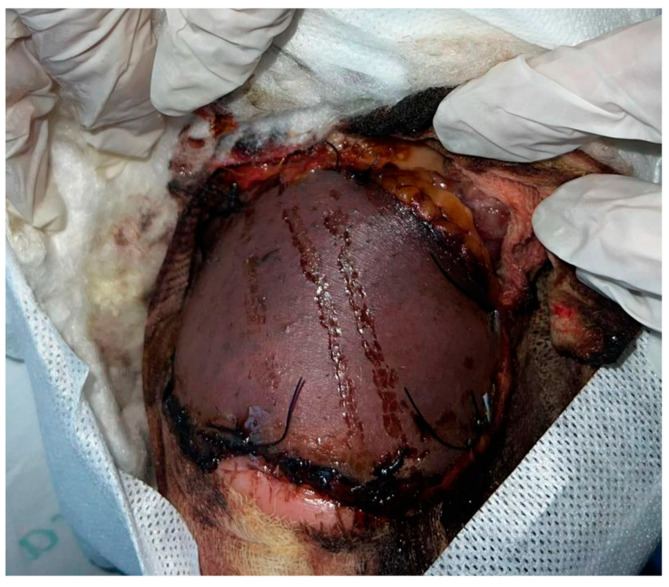
Observation of flap necrosis, 10 days following reconstruction.

Doppler ultrasonography confirmed the presence of peroneal artery perforators. The flap, designed with a 4 cm wide fascial pedicle over the gastrocnemius muscle, was positioned approximately 5 cm above the lateral malleolus, rotated into place, and its vascularity was ensured by observing bleeding before suturing. The flap was not tunnelled to preserve adequate vascularization and prevent vascular compression. However, 10 days following flap reconstruction, the patient exhibited flap hypoperfusion, advancing to 85% necrosis (Figure 3).

**Figure 4:**
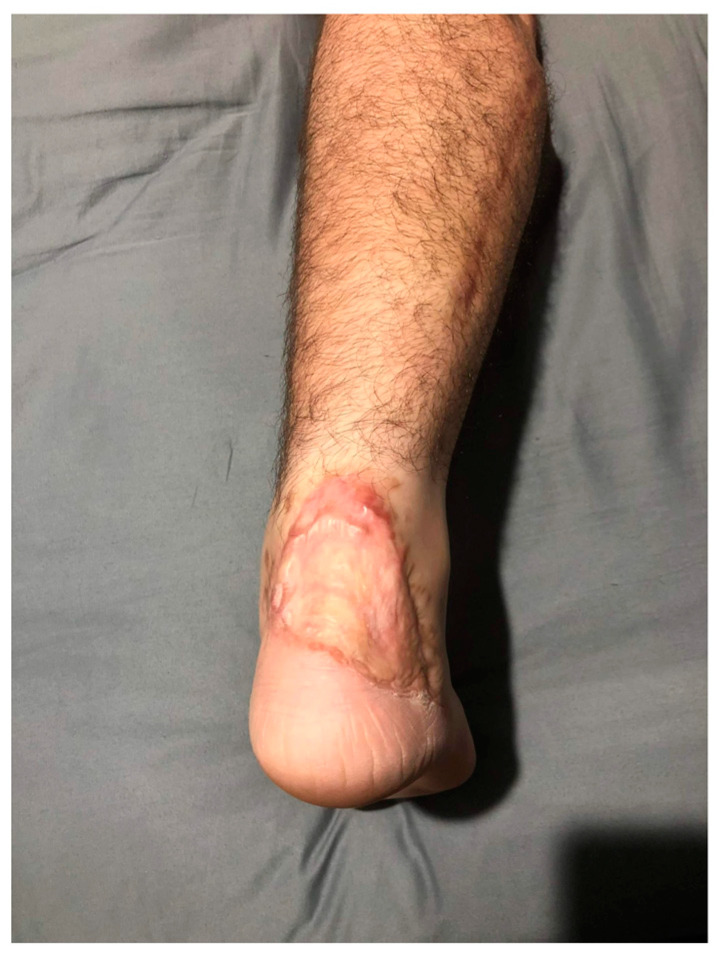
Postoperative outcome 1 year after the procedure.

Subsequent management involved debridement of necrotic tissue and re-application of NPWT. Once the wound presented satisfactory granulation, it was covered with an acellular dermal matrix. Concomitant cutaneous vasculitis onset immediately after flap necrosis led to a lupus-like syndrome diagnosis, evidenced by elevated fibrinogen levels, and positive ANA, anti-Smith, anti-cardiolipin, and anti-beta 2 glycoprotein I autoimmunity markers, which contributed to thrombosis and the flap’s necrosis. Following cortisone and antiplatelet therapy, the patient underwent a second debridement operation and soft tissue coverage with an acellular dermal matrix. Fifteen days later, a third surgery involved the placement of a dermoepidermal graft from the right thigh to address the substance loss. One-year postoperative follow-up is documented in Figure 4, which shows good cosmetic and functional postoperative outcomes.

## 3. Discussion

Managing soft tissue defects in the lower extremities, especially around the calcaneus and external malleolus, presents a substantial challenge to reconstructive surgeons. The case of a 20-year-old male, who sustained injuries from a motor vehicle accident leading to a 7 × 7 cm^2^ soft tissue defect and Achilles tendon rupture, highlights the complexity of treating such injuries. Immediate debridement and soft-tissue coverage with a skin graft, followed by NPWT, initially seemed promising. However, the subsequent necrosis and infection necessitated further surgical intervention, employing the reverse sural flap technique for soft tissue reconstruction. The onset of lupus-like syndrome, diagnosed through various autoimmune markers, introduces an additional layer of complexity to this case. The autoimmune condition likely contributed to thrombosis and the flap’s necrosis, illustrating the critical role of systemic factors in flap viability and wound healing. This aspect emphasizes the need for comprehensive preoperative assessment and consideration of systemic conditions that may affect surgical outcomes. The prothrombotic pathophysiology of SLE significantly elevates the risk of thrombotic events, which can compromise the delicate blood supply to surgical flaps, leading to ischemia and tissue necrosis [10]. Therefore, venous thromboembolism (VTE) prophylaxis is critical in optimizing surgical duration and non-pharmacological interventions like compression stockings [10,11]. Effective post-operative management of thromboses, including low-molecular-weight heparin and nitroglycerin, is equally crucial [10,12,13,14,15]. Robertson et al. found that topical nitroglycerin can reduce the incidence of mastectomy flap necrosis postoperatively [9]. Systematic reviews by Boissiere et al. and Herlin et al. have highlighted the effectiveness of leech therapy as a viable strategy to reduce venous congestion in flaps [13,14]. Odorico et al. and Shen et al. emphasize the importance of meticulous flap monitoring to prevent failure, underlining its significance in reconstructive surgery outcomes [16,17]. Furthermore, Dang et al. have identified infrared thermography as a potentially valuable tool [3,18]. However, they note that the scarcity of available evidence limits the reliability of their findings, suggesting a need for further research in this area. This underscores the multifaceted approach needed for patient safety and flap viability in plastic surgery. The distally based sural flap is the workhorse flap for the reconstruction of the lower leg, the ankle, and the foot. Another alternative reconstructive option includes a free flap. There is ongoing debate among reconstructive surgeons about whether the free flap is better than a sural flap. When considering the reconstructive ladder, reverse sural flap, as a locoregional flap, is preferable as a reconstructive option. The greatest advantage of a sural flap is its simplicity, allowing even non-plastic surgeons to perform it easily and rendering it advantageous in resource-poor centres [17]. Tripathee et al. conducted a systematic review encompassing 2575 patients across 89 articles over 19 years, reporting an overall complication rate of 25.20%, with partial flap loss being the most common complication at 7.85% [14,16,17,18,19,20,21,22,23,24,25,26,27,28,29,30,31,32,33,34,35,36,37,38,39,40,41]. This extensive pooled analysis underscores the relative reliability and highlights significant room for improvement in outcomes related to reverse sural flaps [12,13,22,23]. An observational study by Clivatti GM et al. on nine reverse-flow fasciocutaneous sural flaps reported four cases with partial necrosis, indicating variability in success rates potentially due to patient-specific factors or surgical technique nuances [9,13,14,15,16,17,18,19,20,21,22,23,24,25,26,27,28,29,30,31,32,33,34,35,36,37,38,39,40,41,42]. Cho ÁB et al.’s prospective cohort study on modified sural flaps with covered pedicles reported satisfactory outcomes without flap loss among their twenty cases, suggesting modifications can reduce complication rates compared to conventional techniques [9,14,15,16,17,18,19,20,21,22,23,24,25,26,27,28,29,30,31,32,33,34,35,36,37,38,39,40,41,42]. Yammine K et al.’s systematic review specifically evaluated the effectiveness of reverse sural flaps in diabetic foot ulcers among 187 patients across 33 studies [25]. They found a high healing rate but acknowledged that RSF might be less successful in diabetic wounds than trauma-induced ones due to inherent patient-related risk factors. Ciofu RN et al.’s work, Schmidt K et al.’s literature analysis, and Özkan HS’s study on RSA flaps in head and neck reconstruction, Chauhan VS’s examination into plaster burns treated by reverse sural flap, Grandjean A’s pediatric-focused study on distally based sural flaps for ankle and foot coverage each contribute unique insights into specific applications or modifications that could potentially influence outcomes [14,20,21,22,23,24,25,43]. These depend on context-specific variables, such as the patient’s age, or underlying conditions like diabetes mellitus or peripheral vascular disease affecting wound healing capabilities [21,22,26,27,28,29]. In 2003, Baumeister et al. clarified that the RSA flap yielded a higher rate of necrosis in high-risk, critically multimorbid, and older patients [26]. Karacalar et al.’s standard bi-pedicled delay procedure enhanced flap perfusion by dilating the arterial network but did not demonstrate increased vascular neogenesis [35]. We hypothesized that delaying the flap (the delay time ranged from 48 h to 2 weeks) and using a 4 cm wide pedicle would decrease the amount of partial flap necrosis that commonly occurs with this flap using the previously described techniques [35,36,37]. Significant factors contributing to tissue necrosis, such as severe ischemia, are more prevalent in the lower extremity. This prevalence underscores an urgent need for effective treatments for chronic wounds in the leg, ankle, and heel regions. The collective body of research emphasizes several key points: the adaptability and utility of reverse sural flaps in various clinical contexts, a recurring theme that suggests technique modifications can increase flap viability and decrease the risk of complications, and the critical importance of thorough preoperative planning, especially in assessing patient comorbidities that greatly influence the success of the outcomes [38]. Challenges persist despite its benefits, such as esthetic considerations at the donor site and the preservation of major vessels. Venous congestion often leads to partial or complete flap losses, necessitating further interventions [34]. Through guided secondary healing processes, these interventions may be surgical, such as skin grafting, flap revisions, or conservative. These challenges highlight the necessity for technical expertise and comprehensive postoperative management strategies to ensure optimal recovery paths. The existing literature on reverse sural flaps establishes this method as a generally reliable approach for soft tissue reconstruction within certain anatomical areas [35,39,40]. However, it also comes with notable risks of complications, primarily partial or complete flap losses, primarily due to venous congestion. Other issues include infection rates and unsightly scarring at the donor site, which may require further corrective actions. This underscores the ongoing need for surgical innovation, the adoption of modifications, and improved perioperative care protocols. Such efforts aim to reduce the incidence of adverse events and elevate the overall effectiveness of reconstructive surgery practices.

## 4. Conclusions

The reverse sural flap represents a valuable method for addressing lower limb defects, offering a viable pathway for reconstruction with considerations for esthetic outcomes and vessel preservation. However, its susceptibility to necrosis underscores the necessity for meticulous preoperative assessments, including thorough evaluations of vascularization, autoimmune profiles, and potential cardiovascular conditions. Beyond these measures, management strategies for necrosis should include vigilant post-operative monitoring, timely intervention at the first signs of compromised vascular supply, and adopting adjunct therapies to enhance flap viability. Such comprehensive evaluations are crucial to optimizing the procedural success of reverse sural flap reconstructions. The following highlights the key points of consideration:

- Utility of the reverse sural flap: it is a valuable technique for addressing lower limb defects, offering good esthetic outcomes and preserving vital vessels;
- Risk of necrosis: the flap is susceptible to necrosis, making thorough preoperative assessments essential;
- Preoperative evaluations: these should include careful assessment of vascularization, autoimmune profiles, and cardiovascular conditions;
- Necrosis management: postoperative monitoring should be vigilant, with timely intervention at the first sign of compromised vascular supply.

## Data Availability

No new data were created or analyzed in this study. Data sharing is not applicable to this article.
